# *Leishmania major* Cutaneous Leishmaniasis in 3 Travelers Returning from Israel to the Netherlands

**DOI:** 10.3201/eid2211.161154

**Published:** 2016-11

**Authors:** Justin S. Kuilder, Pieter J. Wismans, Ewout M. Baerveldt, Jaap J. van Hellemond, Mariana de Mendonça Melo, Perry J.J. van Genderen

**Affiliations:** Harbour Hospital, Rotterdam, the Netherlands (J.S. Kuilder, P.J. Wismans, E.M. Baerveldt, J.J. van Hellemond, M. de Mendonça Melo, P.J.J. van Genderen);; Erasmus University Medical Center, Rotterdam, the Netherlands (E.M. Baerveldt, J.J. van Hellemond)

**Keywords:** leishmaniasis, cutaneous, Israel, travel, vector-borne diseases, Leishmania major, parasitic diseases, protozoa

**To the Editor:** Cutaneous leishmaniasis (CL) is a protozoan disease transmitted by sand flies that usually runs a relatively mild course. Classic CL starts as a red papule at the place of the insect bite; it gradually enlarges into a painless nodule or plaque-like lesion, which eventually becomes encrusted. When the crust falls off, a typical ulcer with raised and indurated border becomes apparent. CL can cause considerable illness and may leave disfiguring and disabling scars after healing. The interplay between *Leishmania* species and host immune response is complex, and, as a result, disease manifestations may vary substantially among species as well as among infected persons (*1**,**2*). An estimated 0.7–1.2 million new CL cases occur annually in tropical and subtropical regions of the world. CL is currently endemic in >98 countries worldwide; Afghanistan, Algeria, Colombia, Brazil, Iran, Syria, Ethiopia, North Sudan, Costa Rica, and Peru together account for up to 75% of global estimated CL incidence (*3*).

We report 3 travel companions from the Netherlands who all acquired CL after they participated in a short-term study course in Israel during September–October 2015. The travelers visited several places in the Negev Desert in southern Israel. All cases were confirmed by PCR with additional sequence analysis of the mini-exon locus and the 3′ untranslated region of the HSP70 locus, demonstrating *L. major* as the causative species (*4*).

The first case-patient was a 55-year-old man who observed red papules on his head and shoulder 1 month after he returned to the Netherlands. Gradually, these papules increased in size, and number and showed a tendency to ulcerate. On examination at the Institute for Tropical Diseases in Rotterdam, 12 painless, hyperkeratotic, plaquelike, sharply demarcated lesions were identified, some partially ulcerated, located on the head, shoulders, arms, legs, and across the chest. Histopathologic examination on skin biopsy specimens acquired from 1 lesion revealed *Leishmania* amastigotes, consistent with a diagnosis of CL. The patient was treated with miltefosine (50 mg orally 3×/d for 28). Clinical recovery followed gradually.

The second case-patient, a 52-year-old woman, noticed some red papules on both legs that gradually increased in size and ulcerated in the 2 months after return to the Netherlands. She was initially treated by a general practitioner for a presumed bacterial skin infection but did not show a clinical response to antibiotic treatment. On examination, also at the Institute for Tropical Diseases, 6 painless ulcers were seen on her legs. CL was suspected after taking into account the clinical manifestations and the recent diagnosis of CL in her travel companion. She was successfully treated with miltefosine after the diagnosis was confirmed.

A third case-patient, a 52-year-old woman, was diagnosed with CL after she sought treatment for a single small, sharply demarcated, painless pretibial plaquelike skin lesion on her arm that had been present for 2 months after her return to the Netherlands. Repeated PCRs of skin biopsy specimens confirmed the diagnosis of *L. major* CL. She preferred a “wait and see” policy over treatment.

The 3 patients with CL, a cluster of travel companions, were conceivably infected in the Negev Desert. Only 1 previous report has documented a traveler returning from Israel who was diagnosed with CL at the Institute for Tropical Diseases during 2007–2016 (Table). Most cases originated from the New World, in particular from South America, followed by Central America (Table). Few of these cases originated from the Old World, where most of the global effects of CL occur. CL is more frequently diagnosed among long-term travelers such as military personnel, adventure travelers, photographers, and researchers, rather than among short-term travelers (*5*).

**Table:** Characteristics of 3 travel companions to Israel and a cohort of patients with imported cutaneous leishmaniasis previously diagnosed at the Institute for Tropical Diseases, Harbour Hospital, Rotterdam, the Netherlands, 2007–2016

Characteristics	Case-patient 1	Case-patient 2	Case-patient 3	Cohort, n = 27*
Age, y (SD)	55.2	52.6	52.3	45.4 (15.2)
Sex	M	F	F	M: 19 (70.4); F: 8 (29.6)
New World/Old World	Old World	Old World	Old World	New World: 18 (66.7); Old World: 9 (33.3)
Country of acquisition	Israel	Israel	Israel	Argentina: 1 (3.7); Brazil; 2 (7.4); Costa Rica: 2 (7.4); Ecuador: 3 (11.1); Ethiopia: 1 (3.7); French-Guyana: 1 (3.7); Israel: 1 (3.7); Mexico: 1 (3.7); Morocco: 4 (14.8); Peru: 1 (3.7); Spain: 2 (7.4); Suriname: 6 (22.2); Trinidad and Tobago: 1 (3.7); Turkey: 1 (3.7)
Reason for visit	Tourism	Tourism	Tourism	Tourism: 18 (66.7); work-related visit: 2 (7.4); family visit: 6 (22.2); unknown: 1 (3.4)
Clinical manifestation	Ulceration/plaque-like lesion	Ulceration	Plaque-like lesion	Erythematous papule: 1 (3.7); nodule/plaquelike lesion: 9 (33.3); ulceration: 17 (63.0)
Mean no. skin lesions	12	6	1	1.6 (median 1.0, range 1.0–5.0)
Localization	≥1 localization	≥1 localization	Lower leg	Face: 4 (14.8); upper arm: 3 (11.1); lower arm: 10 (37.0); upper leg: 0; lower leg: 6 (22.2); trunk or neck: 1 (3.7); ≥1 localization: 3 (11.1)
Diagnostic method	Histology/PCR	Histology/PCR	Histology/PCR	Histology: 0; PCR: 19 (70.3); histology/PCR: 8 (29.7)
*Leishmania* spp.	*L. major*	*L. major*	*L. major*	*L. major*: 2 (7.4); *L. tropica*: 1 (3.7); *L. infantum*: 4 (14.8); *L. donovani*: 1 (3.7); *L. mexicana*: 1 (3.7); *L .braziliensis*: 7 (25.9); *L. guyanensis*: *4* (14.8); *L. panamensis*: 1 (3.7); *L. lainsoni*: 1 (3.7); Unknown: 5 (18.5)
Treatment	Miltefosine	Miltefosine	Wait and see	Cryotherapy: 2 (7.4); topical paromomycin/methylbenzethoniumchloride: 1 (3.7); theurapeutic excision: 1 (3.7); fluconazole: 1 (3.7); pentamidineisethionate: 2 (7.4); systemic stibogluconate: 3 (11.1); miltefosine: 17 (63.0)

*Values are no. (%) except as indicated.

Recent reports have described a 7-fold increase in *L. major* CL cases among inhabitants of the Negev Desert (*6*), with urban expansion into CL-endemic foci and changes in land use currently regarded as the most probable causes for this increase in incidence (*6**,**7*). A concurrent increase among travelers has not occurred so far, although the aforementioned cases might indicate that the increasing risk of contracting CL in the Negev Desert is not only restricted to its inhabitants but may also pose a risk to short-term travelers.

The spectrum of disease of CL is highly variable, even among persons infected with the same *Leishmania* species (*1**,**2**,**8*), as illustrated by this cluster of *L. major* CL cases among persons who traveled together to Israel. Because *L. major* CL might mimic other infectious and inflammatory diseases, physicians assessing travelers with painless and persistent skin ulcers after their return from CL-endemic countries should therefore consider CL in their differential diagnosis. In conclusion, awareness should be raised among physicians and healthcare workers that CL is not exclusively limited to tropical countries but may also be acquired by short-term travelers to more temperate regions, such as southern Europe and the Levant (Syria, Lebanon, Israel, and Jordan) (*3*,*9*).
